# Epigenetic Control of Pancreatic Regeneration in Diabetes

**DOI:** 10.3390/genes9090448

**Published:** 2018-09-07

**Authors:** Shruti Balaji, Tiziana Napolitano, Serena Silvano, Marika Elsa Friano, Anna Garrido-Utrilla, Josipa Atlija, Patrick Collombat

**Affiliations:** Université Nice Sophia Antipolis, Inserm, CNRS, iBV, FR-06100 Nice, France; sbalaji@unice.fr (S.B.); tiziana.napolitano@unice.fr (T.N.); serena.silvano@unice.fr (S.S.); marika-elsa.friano@unice.fr (M.E.F.); anna.garrido-utrilla@unice.fr (A.G.-U.); josipa.atlija@unice.fr (J.A.)

**Keywords:** epigenetics, pancreas, regeneration, diabetes

## Abstract

Both type 1 and type 2 diabetes are conditions that are associated with the loss of insulin-producing β-cells within the pancreas. An active research therefore aims at regenerating these β-cells with the hope that they could restore euglycemia. The approaches classically used consist in mimicking embryonic development, making use of diverse cell sources or converting pre-existing pancreatic cells. Despite impressive progresses and promising successes, it appears that we still need to gain further insight into the molecular mechanisms underlying β-cell development. This becomes even more obvious with the emergence of a relatively new field of research, epigenetics. The current review therefore focuses on the latest advances in this field in the context of β-cell (neo-)genesis research.

## 1. Introduction

The pancreas is an organ that contains both an exocrine and an endocrine compartment, all of its cells arising from a common epithelium [1]. The exocrine compartment secretes and transports digestive enzymes to the duodenum, whereas the endocrine tissue is organized into scattered clusters of cells, termed islets of Langerhans. The latter contain five different hormone-secreting cell subtypes: α-, β-, δ-, ε-, and pancreatic polypeptide (PP)-cells producing glucagon, insulin, somatostatin, ghrelin and PP, respectively. Among these different hormones, insulin acts to decrease blood sugar levels, whereas glucagon prevents hypoglycemia. 

Diabetes is a condition that results from an alteration of insulin production or action. It is divided into two major types: Type 1 diabetes (T1D) corresponds to an autoimmune disorder wherein the β-cells are selectively destroyed whereas type 2 diabetes (T2D) is caused either by a loss of function of the β-cells or a resistance to insulin action in the peripheral target tissues (liver, muscle, adipose, etc.). Both forms of diabetes eventually result in β-cell loss and chronic hyperglycaemia [2]. Concerning T1D, while current therapeutic approaches (insulin supplementation, islet transplantation) save lives, the overall life expectancy of diabetic patients remains decreased, and their quality of life remains altered [3,4,5]. Numerous research laboratories therefore attempt to develop alternative therapies. Most of these aim at (re-)generating a functional insulin-producing β-cell mass by recapitulating endocrine pancreas development, as seen during embryogenesis [6,7,8,9,10]. Such an approach could also be useful for late-stage T2D patients, as these display β-cell loss as the disease progresses. However, it is clear that we need to gain further insight into the molecular mechanisms underlying β-cell development prior to an eventual application. This is mostly due to the fact that pancreas morphogenesis is a highly dynamic process that requires a precise pattern of activation/inactivation of several genes, quite often in a concomitant fashion. 

In addition to this intricate genetic control, epigenetic regulation adds an additional layer of complexity. Epigenetics, as elegantly defined by others, corresponds to “the structural adaptation of chromosomal regions so as to register, signal or perpetuate altered activity states” [11]. Since chromatin consists of DNA wrapped around histone proteins, epigenetics also refers to DNA methylation and histone modifications that modulate gene expression without affecting the nucleotide sequence itself [12,13]. Several studies and reviews have been dedicated to the understanding of this relatively new field with a focus on the determination of whether particular regions of the genome are in an opened or closed state [11,14,15]. Indeed, chromatin that is not compacted (opened) allows for genes to be transcribed, while the opposite favors the silencing of genes. Opened chromatin marks include de-methylated DNA, histone methylation (H3K4me1/2/3), histone de-methylation (H3K27), and histone acetylation (H3K4/27ac). On the other hand, DNA methylation, histone methylation (H3K27me3), and histone deacetylation represent hallmarks of closed chromatin. The proteins that bring about these changes, as well as those that recognize the chromatin modifications at a specific genomic locus and regulate target gene expression by the recruitment of the required transcription factors (TFs), are known as epigenetic regulators. The histone modifying enzymes, long non-coding RNAs (lncRNAs), and chromatin status readers, such as bromodomain-containing proteins (BET) and lethal 3 MBT-like protein-1 (L3MBTL1), are examples of epigenetic regulators [16,17]. 

In the context of the pancreas, since all endocrine cells arise from a common progenitor pool where a balance of activation/inactivation of several concomitantly expressed TFs determines cell allocation, the role of epigenetics and epigenetic regulators is crucial. From a pharmacological and organ regeneration perspective, this may be even more important than attempting to control gene expression. The current review therefore aims at summarizing the advances that were made in recent years in the field of epigenetics with particular emphasis on the mechanisms underlying pancreatic β-cell (neo)genesis.

## 2. Advances in Our Understanding of the Epigenetics of Pancreas Development

The first morphological signs of pancreas development are detected at around embryonic day 9.5 (e9.5) in mice [1]. At this stage, the endoderm evaginates into the overlying mesenchyme, forming the dorsal bud. This is followed by the budding of the ventral pancreas under the control of a different molecular cascade. The two buds eventually fuse at around e12–e13 to form a single organ, the pancreas. Concomitantly, the process of endocrine specification is initiated, as outlined by the expression of several TFs, including Pancreas/duodenum homeobox protein 1 (*Pdx1*), Neurogenin 3 (*Ngn3*), Aristaless-related homeobox (*Arx*), and Paired box gene 4 (*Pax4*). While Pdx1 labels the early pancreatic progenitors, Ngn3 is a marker of pancreatic endocrine commitment [18,19]. *Arx* expression restricts the endocrine-committed cells to an α-cell fate, while *Pax4* expression guides cells towards a β- or δ-cell lineage [8,20,21]. In mice, the proliferation of endocrine cells and their compaction into islets are known to continue for a few days postpartum. 

**Figure 1:**
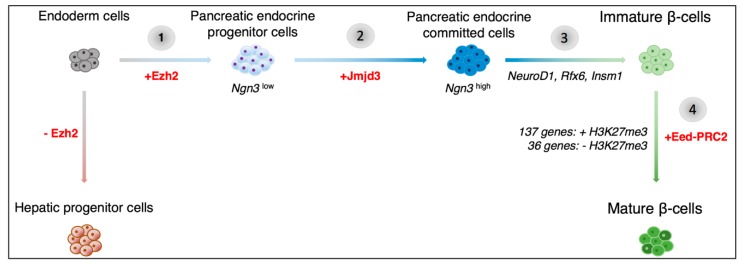
Schematics of the potential epigenetic mechanisms involved in pancreatic β-cell development. Previous research has shown that the transition from endoderm cells to pancreatic progenitor cells involves the activation of the Enhancer of zeste homolog 2 (Ezh2) histone methyltransferase (**1**). Subsequently, Neurogenin 3 (*Ngn3*)^low^ pancreatic endocrine progenitor cells transition to a *Ngn3*^high^ cell state and are thereby committed to the endocrine cell lineage (**2**). The histone demethylase, Jumonji domain-containing protein 3 (Jmjd3), was found to be involved in this process. A high level of Ngn3 expression leads to the activation Ngn3 downstream targets, such as Neurogenic differentiation factor 1 (*NeuroD1*), Regulatory factor X, 6 (*Rfx6*), and Insulinoma-associated 1 (*Insm1*) (**3**). The resulting immature β-cells further develop through the silencing of the immaturity-associated genes (via the addition of H3K27me3 marks) and the de novo activation of maturity-associated genes (with the loss of H3K27me3 marks) (**4**). The epigenetic changes associated with this stage in development are regulated by the polycomb repressor complex (EeD-PRC2).

In terms of epigenetics, several studies have previously uncovered the bivalent status of pancreatic regulatory elements in the early developing pancreas, reviewed in [12,13]. Indeed, at early stages, the chromatin within these regulatory regions bears both active and repressive histone marks. The choice between the maintenance or loss of a given mark is made based on both inter- and intra-cellular developmental cues. Interestingly, Xu et al. have previously shown that the Polycomb repressor complex 2 (PRC2) protein, Enhancer of zeste homolog 2 (Ezh2), plays a key role in assigning a ventral pancreatic fate versus a hepatic fate in endodermal cells, Figure 1 [22,23]. By adding repressive H3K27me3 marks to Pdx1 regulatory elements, Ezh2 maintains the balance between hepatic and pancreatic progenitor cell numbers [22]. The addition of the inactive H3K27me3 marks specifically by the polycomb repressor proteins, has been shown to occur in a step-wise manner [24]. Pancreatic progenitor cells are thus progressively programmed towards a particular endocrine cell fate.

Recent work by Yu et al. allowed us to gain further insight into the Ngn3-induced transition of endocrine progenitor cells to endocrine-committed cells [25]. Neurogenin 3 is a basic helix-loop-helix-containing transcription factor whose targeted disruption leads to a lack of pancreatic endocrine cells in mice [18]. Yu et al. confirmed that, in the pancreatic context, the levels of Ngn3 directed cell fate [25]. A low level was associated with the maintenance of the pluripotent state, whereas a high level of *Ngn3* expression was found to induce endocrine cell lineage allocation, Figure 1. 

Interestingly, the transition from a *Ngn3*^low^ to *Ngn3*^high^ state was associated with the resolution of bivalency within the promoter region of 590 out of 2131 genes initially harboring bivalent marks. Interestingly, for 80% of these genes, bivalency was resolved by the sole loss of H3K27me3 repressive marks, while retaining the H3K4me3 active marks, in a Jumonji domain-containing protein 3 (Jmjd3)-dependant process, Figure 1. This is not surprising as Jmjd3 is a known H3K27me3-specific demethylase [26]. Ribonucleic acid-sequencing (RNA-seq) experiments subsequently demonstrated a concomitant increase in Ngn3 downstream target genes expression, such as Neurogenic differentiation factor 1 (*NeuroD1*), Regulatory factor X, 6 (*Rfx6*), and Insulinoma-associated 1 (*Insm1*), providing evidence of the key role exerted by chromatin regulation on the programming of pancreatic precursors towards an endocrine cell fate, Figure 1. 

Several reports have suggested that mature β-cells can be subdivided into different populations that are based on the differential expression of marker genes or cell-surface antigens [27,28]. These distinct cell subtypes could represent differentially maturing and/or functioning β-cells. Interestingly, very little is known about the pancreatic α-cell maturation process. Qiu and colleagues therefore used single-cell RNA-seq to dissect the maturation mechanisms of α- and β-cells [29]. It was found that while α-cells mature primarily through the downregulation of genes expressed in immature α-cells, β-cell maturation involves the upregulation of additional genes, Figure 1. Interestingly, Assay for Transposase-Accessible Chromatin with high-throughput sequencing (ATAC-seq) data from mature α- and β-cells showed that 78% of open chromatin peaks found in β-cells could also be observed in α-cells, whereas only 46% of peaks detected in α-cells were also found in β-cells [30]. Additional work indicated that the difference between mature α- and β-cells may come down to less than 21% of active genes. Since Qiu et al. suggested that α-cell maturation involves the silencing of certain genes, while β-cell maturation involves the activation of new genes, one would assume that more open chromatin peaks would be found in mature β-cells as compared to α-cells [29]. However, Ackermann et al. found that nearly 27,000 ATAC-seq peak regions were classified as α-cell-specific, whereas only 1850 β-cell-specific regions were detected [30]. What is even more interesting is that combined with mRNA-seq data, it was suggested that open chromatin may be a better predictor of gene activation in mature α-cells than in β-cells, thus implying that differential regulation alone could explain the phenotypic and functional difference between the two cell subtypes. 

Adding further to our understanding of the differential allocation to the various endocrine cell lineages, Neiman et al. studied the methylation pattern of CpG islands within the promoter region of various β-cell specific genes [31]. Surprisingly, while they found that the promoters of β-cell specific genes were highly methylated in non-endocrine tissue, such as in acinar cells, this difference was not visible when comparing the different endocrine populations themselves. Indeed, the *INS* and *GCG* gene promoters were found unmethylated in Fluorescence-activated cell sorting (FACS) sorted α-, β-, and δ-cells from human islets. However, the methylation of CpG sites downstream of the transcription start sites (TSS), i.e., at the enhancers, reflected the differential gene expression pattern amongst the endocrine subtypes. Methylation downstream of the *INS* TSS was thus found exclusively in α- and δ-cells, whereas methylation downstream of the *GCG* TSS was found in β- and δ-cells. Further, it appeared that such a methylation pattern was obtained progressively with age. Indeed, prior to birth, all Ngn3-positive progenitors displayed the same demethylation pattern. These observations are of interest as they further outline the cellular plasticity that is often observed in pathological conditions, as well as in attempts to transdifferentiate alternative endocrine cell subtypes into β-cells.

## 3. The Epigenetic State in Type 2 Diabetes and Obesity

Type 2 diabetes is caused either by a loss of insulin secretion or insulin resistance. Interestingly, recent in-depth analyses of the mouse and human genome by Lu et al. showed that in mice subjected to a high fat diet (HFD) and in humans with T2D, chromatin-state-defined transcriptome dysregulation led to a preferential loss of key β-cell TFs [32]. Equally important was the observation that such loss of identity was associated with a transition to a bivalent epigenetic state that is normally associated with stemness, suggestive of dedifferentiation [32]. This is in accordance with the finding that humans with a high BMI display a decline in β-cell function [33]. Interestingly, an embryonic ectoderm development protein (Eed)-containing PRC2 was shown to be required for the long-term maintenance of β-cell function in vivo. The Eed protein along with the Ezh2 histone methyltransferase and other proteins, form the PRC2. The PRC2 catalyzes the trimethylation of H3K27, thus leading to target gene repression. The loss of the Eed-PRC2 complex was found to result in the expression of islet dedifferentiation factors, like GLI-Kruppel family member 2 (*Gli2*), and in the loss of the terminal β-cell identity [32]. Importantly, the simultaneous inhibition of a polycomb opposer, such as *MII* (a H3K4 methyltransferase), could rescue this loss of a β-cell phenotype, thereby raising hope in the context of T2D research. Equally noteworthy was the finding that multiple T2D risk loci are, in fact, α-cell-specific, as demonstrated while using ATAC-seq, further suggesting that T2D may also be associated with α-cell dysfunction and not just β-cell failure [30].

A few years ago, mutations within the high mobility group (HMG) box-containing protein 20A (*HMG20A*) gene were reported in T2D populations [34,35]. This protein is a known chromatin modifier in neurons, but it was not until recently that its role in the maintenance of pancreatic β-cell function was reported [36]. It has an opposing role to another chromatin modifier, RE-1 silencing transcription factor (Rest) [37]. Whereas Rest is completely absent in mature islets, HMG20A is expressed in islets from normal individuals. There, HMG20A normally represses β-cell development-associated genes, like *Pax4* and *NeuroD1*, and instead activates β-cell maturation-associated genes, such as v-maf musculoaponeurotic fibrosarcoma oncogene family, protein A (*MafA*) [36]. Mutations in *HMG20A* that are associated with T2D could thus alter its function in repressing the ‘disallowed’ genes and result in impaired glucose-stimulated insulin secretion (GSIS) and other functional markers of mature β-cell function. This could occur by the deposition of active H3K4me2 marks at the promoters of genes, such as *Pax4* [36]. 

Upon a glucose bolus, β-cells sense glucose levels in the systemic circulation via their Glucose transporter 2 (Glut2 receptors) [38], which eventually triggers the exocytosis of insulin. Insulin then mobilizes peripheral tissues, viz. liver, fat and muscle, to take up glucose thereby leading to a reduction of glucose in the peripheral circulation [39]. Insulin resistance results from the unresponsiveness and/or loss of insulin receptors in peripheral tissues [40,41]. Recently, Kang et al. sought to study the epigenetic basis of insulin resistance, making use of two agents with seemingly opposite actions: Dexamethasone (Dex) and the tumor necrosis factor α (TNF-α), the former being an anti-inflammatory agent and the latter displaying pro-inflammatory properties [42]. Surprisingly, the authors found that treating adipocytes in vitro with either agent resulted in some common epigenetic changes. For instance, an increase in opened-chromatin H3K27ac marks in the distal enhancers of common target genes, viz. Transmembrane protein 176a/b (*Tmem176a/b*) and Family with sequence similarity 46 member b (*Fam46b*), was noted and associated with a decrease in insulin-stimulated glucose uptake. This suggests that although the two agents could act via different signaling pathways, they eventually converge towards a common downstream target. Even more surprisingly, a number of common enhancer sites were found to be bound by the glucocorticoid receptor (GR), previously thought only to be activated by Dex [42]. Accordingly, the expression of some of the common insulin resistance-associated genes, including Vitamin D receptor (*Vdr*), was found to be reduced by treating cells with Rosiglitazone, which is normally used as an insulin-sensitising agent in T2D. Thus, in addition to providing an epigenetic basis for insulin resistance, these studies also provided several therapeutic research paths, such as the use of anti-inflammatory agents, insulin-sensitizing agents, GR inhibitors, alone or in combination. 

As previously mentioned, gaining further insight into the role of epigenetic regulators is also an important aspect of studying the epigenetic causes underlying diseases. lncRNAs being one such class of epigenetic regulators, Akerman and colleagues studied gene expression datasets that included human islet samples from donors that were diagnosed with T2D or impaired glucose tolerance (IGT) [43]. They found that the expression of 15 lncRNAs was strongly reduced in T2D islets, whereas 100 lncRNAs were insufficiently expressed in IGT islets when compared to islets from control individuals. In particular, the lncRNA PDX1 locus upstream transcript (*PLUTO*) was found to be among the most markedly downregulated lncRNAs in islets from T2D or IGT donors. By combining several experimental approaches in the human EndoC-βH1 cell line and in dispersed human islets, they established that inhibiting *PLUTO* expression also severely inhibited *PDX1* expression. This was attributed to a loss of contact between the *PDX1* promoter and its enhancer cluster, i.e., a loss of three-dimensional (3D) chromatin conformation. The loss of lncRNA expression resulting in the loss of expression of key β-cell-associated TFs is thus worth looking into in order to uncover the pathogenic basis of non-genetic diabetes.

While more information is available about the genetic and epigenetic modifications that accompany T2D, much is yet to be understood of the epigenetic basis of T1D. In a recent review, Wu et al. dedicated a short paragraph to this topic [44]. They reviewed the available literature and found that in T1D twins, DNA methylation of B lymphocytes could contribute to T1D pathogenesis. They also postulated that the currently available chromatin modifying drugs, such as 5-Aza and Histone deacetylase inhibitors (HDACs), could potentially be of interest for T1D therapy. Other studies in T1D individuals also supported the notion that epigenetic changes in the immune effector cells are responsible for the immune destruction of the pancreatic β-cells [45,46,47,48]. No studies so far have, however, examined the accompanying epigenetic changes, if any, in the β-cells themselves.

## 4. Epigenetics and Pancreatic Regeneration

In the context of T1D research, numerous laboratories aim at regenerating lost insulin-producing β-cells by either mimicking embryonic development or converting pre-existing cells. Several reports suggest that ectopically expressing key pancreatic differentiation factors, such as *Ngn3*, in immature cells could force these to adopt an endocrine fate. Recently, Fontcuberta-PiSunyer et al. described the use of both inhibitors of histone demethylases, as well as ectopic expression of *NGN3* to improve pancreatic differentiation protocols [49]. Interestingly, both the timing and the cellular context seem to influence the robustness of the protocol. Indeed, only the simultaneous inhibition of *Ezh2* and the concomitant activation of *NGN3* led to an activation of Ngn3 downstream target genes in mouse pancreatic (mPac) cells and mouse embryonic fibroblasts (MEFs). The inhibition of *Ezh2* prior to *NGN3* activation was however found to be required in the induced pluripotent stem cell (iPSC) context. Further, although the authors described the increased expression of islet hormone genes, it remains to be determined whether the differentiated cells are functional. Nevertheless, this approach appears as a promising strategy, as confirmed by studies demonstrating that Ezh2 is required for the proliferation of immature β-cells [29].

While it is desirable to find highly proliferative and ethically-sound alternative tissue sources for the mass production of β-like cells, it is also worthwhile to pursue the re- or trans-differentiation of existing pools of pancreatic cells within the patient body. Not only could this avoid the need for surgery, it could also avoid several differentiation steps given the important differences in methylation patterns between non-endocrine and endocrine cell subtypes [31]. In light of this, a study of Qiu et al. highlighted the proliferative capacity of mature β-cells, thus providing ideas for the use of existing/leftover β-cells in diabetes therapy [29]. They found that although both α- and β-cells are most proliferative a few days after birth, the expression of the histone methyltransferase gene *Ezh2* was maintained at a low level even in adult, non-proliferative cells. An activation of the *Ezh2* gene could therefore potentially silence those genes that are associated with α- and β-cell maturation and provide a way for these cells to revert back to the immature, proliferative state [50]. 

As previously mentioned, Arx is a marker required for the α-cell allocation and maintenance of the α-cell phenotype, while DNA methyltransferase 1 (Dnmt1) is a methyltransferase that recognizes hemimethylated CpGs and re-methylates them [20,51,52]. Building up on previous research, Chakravarthy et al. showed that the simultaneous loss of *Dnmt1* and *Arx* could induce a reprogramming of α-cells into β-cells in vivo [53]. The heterogenous population of β-like cells so obtained expressed many of the mature, functional markers of β-cells within just 8 weeks. Further, they were able to show that the reprogrammed α-cells were able to respond to glucose like native β-cells. Their observations were confirmed in T1D patients with five years of diabetes or more. In these individuals, they found a subset of glucagon^+^ cells lacking both *DNMT1* and *ARX* expression and ectopically expressing β-cell markers, including insulin. While these results suggest a putative attempt at regenerating lost β-cells in type 1 diabetic patients, whether this conversion is eventually completed or whether these converted cells are also attacked by the immune system remains to be determined. As the authors point out though, it is likely that the conditional loss of *Arx* and *Dnmt1* alone would be insufficient to reprogram α-cells into β-cells. However, guided by this information, there is a potential to find out additional (epi)genetic factors that may be key for α-cell-mediated β-like cell neogenesis. 

## 5. The Influence of Culture Substrate on Epigenetic Regulation of Differentiation

While it is well known that the culture substrate influences the differentiation process in vitro ,reviewed in [54], the epigenetic basis of this has only recently been investigated. Thus, Pennarossa et al. checked the global DNA methylation status upon culturing mouse dermal fibroblasts that had been treated with an epigenetic eraser 5-azacytidine on a plastic (stiff) and gel (soft) matrix [55]. Interestingly, they found that, while global methylation decreased as expected upon 5-aza treatment, there was a difference in methylation status when comparing cells cultured on the different substrates, with cells grown on the soft matrix exhibiting lower DNA methylation overall. This was accompanied by a decrease in Histone deacetylase 1 (*Hdac1*) and an increase in Histone acetyltransferase 1 (*Hat1*) and Tet methylcytosine dioxygenase 2 (*Tet2*) gene expression. The cells also acquired a dedifferentiated-like state with an increased expression of pluripotency markers, such as Octamer-binding transcription factor 4 (*Oct4*), *Nanog*, and Sex determining region Y-box 2 (*Sox2*). Interestingly, the induction of pancreatic differentiation while using a three-step protocol showed that the cells grown on the soft matrix were more efficiently differentiated down the pancreatic lineage. Indeed, these cells eventually formed islet-like structures that are composed of a heterogenous population of mostly mono-hormonal cells. These islet-like clusters were further able to respond well to a high glucose challenge, thereby opening new research avenues for β-cell differentiation protocols.

## 6. Conclusions

Although a number of pharmaceutical agents are currently available to treat the consequences of β-cell loss in diabetic patients, improved alternative treatments remain required. In order to achieve this goal, an active research is ongoing, spanning from designing insulin/glucagon pumps to improving islet transplantation [4,56]. However, numerous reports have highlighted the need to go beyond the currently available solutions due to their many disadvantages [57]. One such avenue of disease therapy is tissue regeneration. The field of regenerative medicine is abuzz with efforts to differentiate or trans-differentiate cells from the pancreas and unrelated tissues [6,8,9,10,58,59,60]. However, despite impressive progresses, regenerating fully functional β-cells remains challenging [61]. Clearly, a better understanding of the molecular mechanisms underlying pancreas development, including the accompanying epigenetic alterations, is required. 

In this short review, we described some of the latest discoveries that were made in the field of epigenetics, these clearly demonstrating that epigenetic manipulations could represent a new approach in order to induce β-cell neogenesis. While it is clear that much work remains to be done due to the further increased complexity, new research avenues with great potential have certainly been opened. It would also be worthwhile to further focus on the altered epigenetic status of T2D patients to uncover potential therapeutic avenues. Similarly, a thorough analysis of the epigenetic status of β-cells in T1D individuals is also severely lacking and such studies could also provide new research strategies.
